# A highly contiguous genome assembly of *Brassica nigra* (BB) and revised nomenclature for the pseudochromosomes

**DOI:** 10.1186/s12864-020-07271-w

**Published:** 2020-12-11

**Authors:** Kumar Paritosh, Akshay Kumar Pradhan, Deepak Pental

**Affiliations:** 1 Centre for Genetic Manipulation of Crop Plants, University of Delhi South Campus, New Delhi, 110021 India; 2 Department of Genetics, University of Delhi South Campus, New Delhi, 110021 India

**Keywords:** *Brassica nigra*, Genome assembly, Gene blocks, Pseudochromosome nomenclature, Evolution

## Abstract

**Background:**

*Brassica nigra* (BB), also called black mustard, is grown as a condiment crop in India. *B. nigra* represents the B genome of U’s triangle and is one of the progenitor species of *B. juncea* (AABB), an important oilseed crop of the Indian subcontinent. We report the genome assembly of *B. nigra* variety Sangam.

**Results:**

The genome assembly was carried out using Oxford Nanopore long-read sequencing and optical mapping. A total of 1549 contigs were assembled, which covered ~ 515.4 Mb of the estimated ~ 522 Mb of the genome. The final assembly consisted of 15 scaffolds that were assigned to eight pseudochromosomes using a high-density genetic map of *B. nigra*. Around 246 Mb of the genome consisted of the repeat elements; LTR/Gypsy types of retrotransposons being the most predominant. The B genome-specific repeats were identified in the centromeric regions of the *B. nigra* pseudochromosomes. A total of 57,249 protein-coding genes were identified of which 42,444 genes were found to be expressed in the transcriptome analysis. A comparison of the B genomes of *B. nigra* and *B. juncea* revealed high gene colinearity and similar gene block arrangements. A comparison of the structure of the A, B, and C genomes of U’s triangle showed the B genome to be divergent from the A and C genomes for gene block arrangements and centromeric regions.

**Conclusions:**

A highly contiguous genome assembly of the *B. nigra* genome reported here is an improvement over the previous short-read assemblies and has allowed a comparative structural analysis of the A, B, and C genomes of the species belonging to the U’s triangle. Based on the comparison, we propose a new nomenclature for *B. nigra* pseudochromosomes, taking the *B. rapa* pseudochromosome nomenclature as the reference.

**Supplementary Information:**

The online version contains supplementary material available at 10.1186/s12864-020-07271-w.

## Background

U [1] based on his observations and preceding cytogenetic work [2] proposed a model on the relationship of some of the cultivated Brassica species. The model, known as U’s triangle, described the relationship of three diploid species – *B. rapa* (Bra, AA, *n* = 10), *B. nigra* (Bni, BB, *n* = 8), and *B. oleracea* (Bol, CC, *n* = 9) with three allopolyploid species – *B. juncea* (Bju, AABB, *n* = 18), *B. napus* (Bna, AACC, *n* = 19) and *B. carinata* (Bca, BBCC, *n* = 17). Subsequent cytogenetic work on inter-specific and inter-generic hybrids between the Brassica species of the U’s triangle and other taxa in the tribe Brassiceae showed close relationships and the group was described as Brassica coenospecies [3, 4].

Since the early cytogenetic work, major insights have been gained into the evolution of the Brassica species based on the extent of nucleotide substitutions in the orthologous genes belonging to the nuclear [5] and plastid genomes [6–9], analysis of genome synteny using molecular markers [10, 11], in situ hybridizations [12], and genome sequencing [13–16]. The most significant observation is that the three diploid species of the U’s triangle – *B. rapa, B. nigra, B. oleracea,* and the other diploid species belonging to the tribe Brassiceae have originated through genome triplication, referred to as the ***b*** event [5]. Genome triplication was followed by extensive chromosomal rearrangements leading to gene block reshuffling vis-à-vis the gene block order in *Arabidopsis thaliana* (At) [17, 18], and gene fractionation due to a differential loss of genes in the three constituent paleogenomes [19]. The diploid species of the tribe Brassiceae are, therefore, mesohexaploids. It is now accepted that tribe Brassiceae is defined by the ***b*** event; it is, however, not clear whether the ***b*** event happened once or more times. The presence of two plastid lineages [6–9] points to a minimum of two independent ***b*** events [20].

Genome assemblies of *B.rapa* [13], *B. oleracea* [14], *B. napus* [15], and *B. juncea* [16] were first reported using short-read Illumina sequencing. More recent assemblies of these species have used long-read sequencing technologies, either PacBio SMRT (single-molecule real-time) sequencing or Oxford Nanopore Technologies (ONT) [21–23]. Scaffolding has been carried out with optical mapping and/or Hi-C technologies. The most extensive assembly of the B genome has been made available from our recent effort on the genome assembly of an oleiferous type of *B. juncea* variety Varuna with SMRT sequencing and optical mapping [23].

We report here a highly contiguous genome assembly of *B. nigra* variety Sangam, a photoperiod insensitive, short-duration variety, grown under dryland conditions, and used as a seed condiment crop in India. The assembly has been carried out using Nanopore sequencing and optical mapping. Previously reported Illumina short-read sequences and a genetic map of *B. nigra* [23] were used for error correction and assigning the contigs and scaffolds to the eight pseudochromosomes. We compared the structure of the B genome of *B. nigra* (BniB) with the genomes of *B. rapa* (BraA) [21], *B. oleracea* (BolC) [22], and also the B genome of *B. juncea* (BjuB) [23]. We propose a revised nomenclature for the *B. nigra* pseudochromosomes based on maximum homology between the A and B genome pseudochromosomes; the *B. rapa* A genome nomenclature being the reference as it was the first Brassica genome that was sequenced [13].

## Results

### Genome sequencing and assembly

We estimated the size of *B. nigra* Sangam (line BnSDH-1) by using kmer frequency distribution of ~40x Illumina PE reads to be ~ 522 Mb (Supplementary Fig. 1**).** Genome sequencing of the *B. nigra* line BnSDH-1 on the Nanopore MinION platform yielded a total of 8,778,822 reads with an N50 value of ~ 10 kb (Supplementary Table 1). The obtained long-reads provided ~100x coverage of the *B. nigra* genome if we consider the genome size to be ~ 522 Mb. The raw reads were assembled into 1549 contigs with an N50 value of ~ 1.48 Mb using the Canu assembler (Table 1). The total size of the assembled contigs was ~ 515.4 Mb, covering ~ 98% of the *B. nigra* genome. Nanopore contigs were error-corrected with ~100x Illumina PE reads [23] using the Pilon program for five iterative cycles. A total of 124,464 nucleotide errors and 229,767 InDels were corrected. Most of the errors, predominantly present in the non-coding regions, were identified and corrected in the first two cycles (Supplementary Fig. 2). The quality of the error-corrected contigs was ascertained after each cycle using BUSCO scores. At the end of the five correction cycles, 95.4% of the gene models were found to be complete.

**Table 1:** Genome assembly statistics of *B. nigra* (BB, *n* = 8) variety Sangam

Oxford Nanopore	✓	✓	✓
BioNano		✓	✓
Linkage Map			✓
Total assembly size (bp)	515,400,203	-	-
Number of contigs	1,549	-	-
Longest contig (bp)	17,509,570	-	-
N50 contig length (bp)	1,488,221	-	-
Number of scaffolds	-	15	-
Total scaffold size (bp)	-	506,396,041	-
Longest Scaffold	-	115,616,497	-
N50 scaffold length (bp)	-	68,578,869	-
Unscaffolded contigs	-	1,051(partial)	-
Number of pseudochromosomes/LGs	-	-	8
Scaffolds assigned to LGs	-	-	14
Contigs assigned to LGs	-	-	-
Unassigned scaffolds to LGs	-	-	-
Unassigned contigs to LGs	-	-	-
Length of assigned sequences to LGs (bp)	-	-	505,183,631
Length of unassigned sequences to LGs (bp)	-	-	30,296,383
N50 pseudochromosome length (bp)	-	-	63,988,665

Optical mapping was used for finding the misassemblies in the contigs and for assembling the contigs into scaffolds. Two different optical maps, one with DLS (Direct Label, and Stain) technology using the DLE-I enzyme, and with NLRS (Nick, Label, Repair, and Stain) technology using *BssS*I enzyme were developed (for details see Methods). A total of 440 Bionano genome maps with an N50 value of 1.6 Mb were generated with the *BssS*I library; 17 Bionano genome maps with an N50 value of 63.4 Mb were generated with the DLE-I library (For details Supplementary File 1). A hybrid assembly protocol was used, which generated 15 scaffolds with an N50 value of ~ 70.4 Mb covering ~ 506.4 Mb of the genome. One hundred forty-eight contigs were found to contain misassemblies, mostly due to the merger of some of the highly conserved syntenic regions. A total of 1051 unmapped sequence fragments with an N50 value of ~ 36.7 kb, covering ~ 30.4 Mb of the genome, remained unscaffolded.

**Fig. 1:**
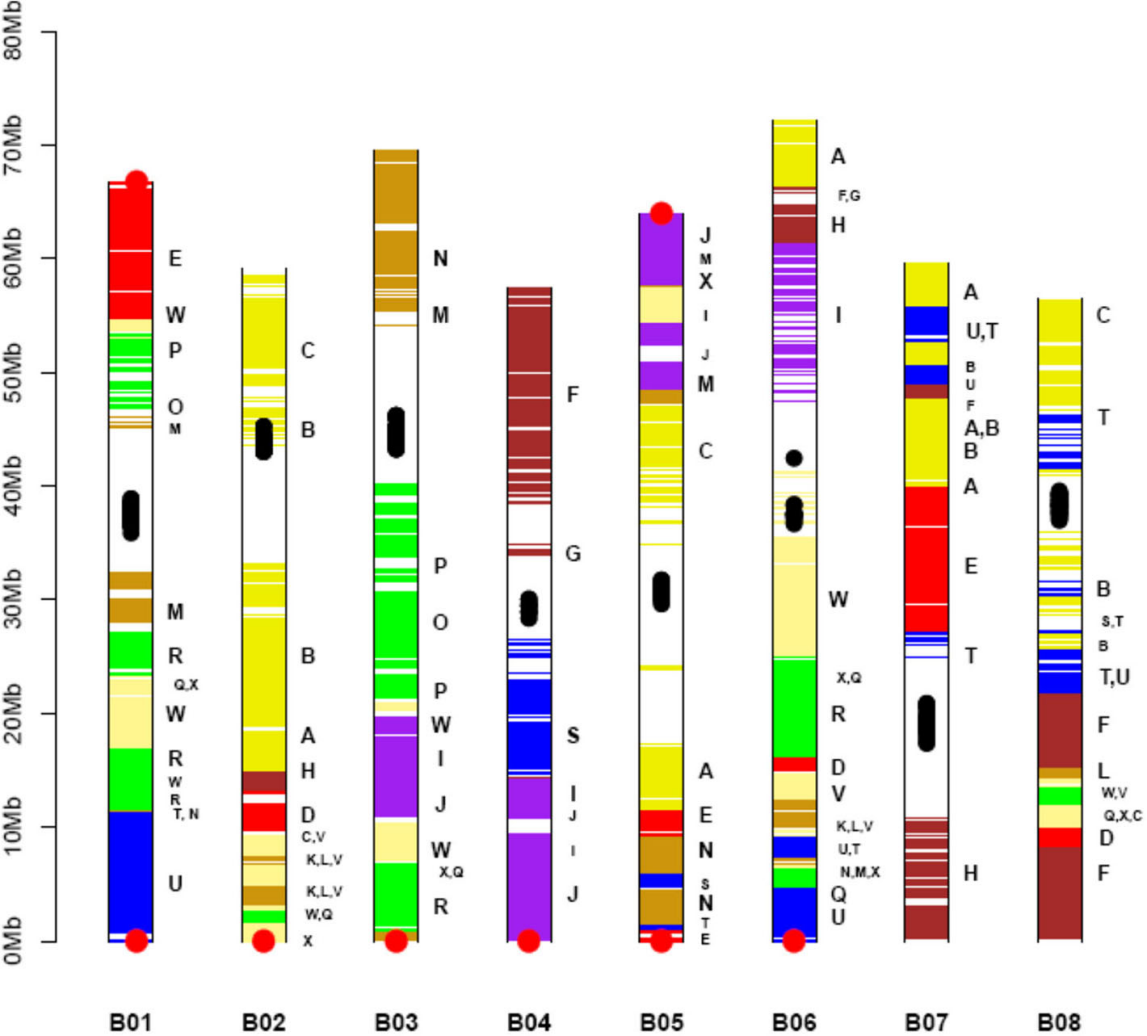
Graphic representation of the *Brassica nigra* pseudochromosomes. Each chromosome is represented by a vertical bar. Each horizontal bar represents a gene. Gene blocks have been identified on the basis of synteny with the *A. thaliana* gene blocks (A-X), as defined and color-coded by Schranz et al. [17]. Centromeric repeats are represented as black dots and telomeric repeats as red dots. A new nomenclature has been given to the *B. nigra* pseudochromosomes on the basis of maximum gene-level collinearity with the *B. rapa* pseudochromosomes [21]

A genetic map of *B. nigra*, with 2723 markers [23], was used to validate the integrity of the scaffolds and to assign these to the eight pseudochromosomes – BniB01 – BniB08 (Fig. 1, Supplementary Fig. 3). The genotyping by sequencing (GBS) based genetic markers were physically mapped on the scaffolds; no misassemblies were observed. Fourteen out of 15 scaffolds could be assembled into eight pseudochromosomes. Five out of the eight chromosomes were represented by a single scaffold each; the remaining three chromosomes consisted of two, three, and four scaffolds (Supplementary Table 2a). One of the scaffolds was found to be unique as no genetic marker mapped on the scaffold; this scaffold consisted of the chloroplast genome of *B. nigra*. The size of the final *B. nigra* genome that could be assigned to the pseudochromosomes was ~ 505.18 Mb (~ 96.7% of the estimated genome size). The current genome assembly provides significantly better coverage than some of the earlier reported assemblies of Brassica species (Supplementary Table 2b).

### Genome annotation for repeat elements, centromeres, and genes

The assembled genome was annotated for the repeat elements, centromeric repeats, and genes. A *de-novo* prediction approach was used for the identification of the TEs. A repeat library was developed following the steps described in the Methods section. *B. nigra* genome contained ~ 246 Mb (47.12%) of repeat elements belonging to three broad categories – DNA transposons, retrotransposons, and other repeat elements. DNA transposons constituted ~ 31 Mb of the assembled genome; ~ 157 Mb of the genome was constituted of retrotransposons. LTR/Gypsy types were found to be the most predominant, ~ 103.1 Mb of the *B. nigra* genome; followed by ~ 43.6 Mb of LTR/Copia types (Supplementary Table 3, Supplementary Fig. 4). LTR/Copia types were found to be most abundant in the vicinity of the centromeric regions. Around 59 Mb of the repeat elements belonged to the unknown repeat category. We earlier carried out a study of the repeat elements constituting the centromeric regions in the B genome of *B. juncea* [23]. The centromere-specific repeats were identified as highly abundant kmers in the putative centromeric regions of the BjuB genome and were characterized for their sequences and their distribution (described in detail in reference [23]); identical repeats were observed to constitute the *B. nigra* centromeric regions (Supplementary Fig. 5).

For gene annotation, the *B. nigra* pseudochromosome level assembly was repeat masked and used for gene prediction with the Augustus program [24] trained with *B. rapa* gene content information. A total of 57,249 protein-coding genes were predicted in the *B. nigra* genome. The predicted genes were validated by comparing these with the non-redundant proteins in the UniProt reference database (TrEMBL); a total of 50,233 genes could be validated at an e-value threshold of 10^− 5^. The predicted genes were further validated by Illumina RNA seq data obtained from the seedling, leaf, and young inflorescence tissues of the line BnSDH-1 and line 2782 (Supplementary File 2). A total of 39,946 genes could be validated by the transcriptome analysis. Transcriptome sequencing was also carried out on the PacBio platform (Supplementary File 2 for all the stats and description). A total of 15,368 full-length *B. nigra* genes were found in the Iso-seq analysis. The Iso-seq analysis validated 2498 additional genes. Thus, a total of 42,444 genes, out of 57,249 predicted genes were validated by the transcriptome analysis of seedling, leaf, and developing inflorescence tissues (Supplementary Fig. 6).

### Gene block arrangement in *B. nigra*

The predicted 57,249 genes in *B. nigra* were checked for their syntenic gene block arrangements by comparisons with the gene block arrangements in the model crucifer At, and the two diploid species of the U’s triangle – *B. rapa* (AA) [21], and *B. oleracea* (CC) [22] with MCScanX. The *B. nigra* genome was divided into 24 gene blocks (A-X), identified in At [17]. Three syntenic regions were identified in the *B. nigra* genome for each gene block in At **(**Supplementary Fig. 7).

Gene fractionation pattern was determined in each of the three *B. nigra* regions syntenic with each of the At gene blocks. Gene retention in the three syntenic regions in *B. nigra* was calculated by taking the number of genes present in the corresponding At gene block as a reference number. Based on the gene fractionation pattern, three sub-genomes were identified in the Bni genome – LF (Least Fragmented), MF1 (Moderately Fragmented), and MF2 (Most Fragmented) (Supplementary Fig. 7). In gene to gene comparison, the LF subgenome was found to contain 10,191 genes, MF1 8822, and MF2 7283 in comparison to a total of 19,091 genes present in the At genome. The three different syntenic regions with differential gene fractionation have been shown earlier to be a characteristic feature of the *B. rapa* and *B. oleracea* genomes [13, 14]. The *B. nigra* genome and the B genome of *B. juncea* reported earlier [23] show a similar pattern of gene fractionation in the three constituent paleogenomes.

The data on the physical position and the expression status of each predicted gene on the eight *B. nigra* pseudochromosomes Bni01 – Bni08 has been provided in Supplementary Table 4. The data contains information on the ortholog of each At gene in the assembled *B. nigra* genome. We carried out the ortholog tagging of each gene of *B. nigra* and identified the nearest ortholog in *B. rapa* (BraA) [21] and *B. juncea* (BjuB) [23] genomes (Supplementary Table 4). A total of 24,799 genes were found to be BniB genome-specific; these could not be found in the syntenic regions of BraA and At genomes. Analysis of the transcriptome data showed 11,503 BniB genome-specific genes to be expressed.

### Comparison of B genome pseudochromosomes of *B. nigra* and *B. juncea*

We compared the B genome assembly of *B. nigra* line BnSDH-1 (BniB) with the B genome assembly of *B. juncea* line Varuna (BjuB) for the gene content, transposable elements, centromeric repeats, and syntenic regions based on gene collinearity. The repeat content in the BniB genome (~ 47.2%) was found to be similar to that in the BjuB genome (~ 51%). The LTR/Gypsy type transposons were the most abundant TEs followed by LTR/Copia types in both the genomes. The distribution of different types of TE elements was found to be similar in both the genomes.

Earlier six B genome-specific repeats were identified in the centromeric regions of the BjuB genome [23]. We found these repeats to be present in a similar manner in the centromeric regions of the *B. nigra* pseudochromosomes (Supplementary Fig. 5) and to be highly identical. In addition, CentBr1, CentBr2, and the other centromeric repeats reported to be present in the BraA, BolC, and BjuA genomes [13, 14, 23] were absent in both the BjuB and BniB genomes. Our analysis indicates that the B genome has undergone a divergent evolutionary path than the A and C genomes in terms of the evolution of the centromeric repeats. The gene number estimation in the BniB genome (57,249) is very similar to the numbers predicted in the BjuB genome (57,084), suggesting no significant loss of genes in the B genome after allotetraploidization. Of a total of 22,498 B genome-specific genes identified in the BjuB genome, 19,175 genes were also detected in the BniB genome.

**Fig. 2:**
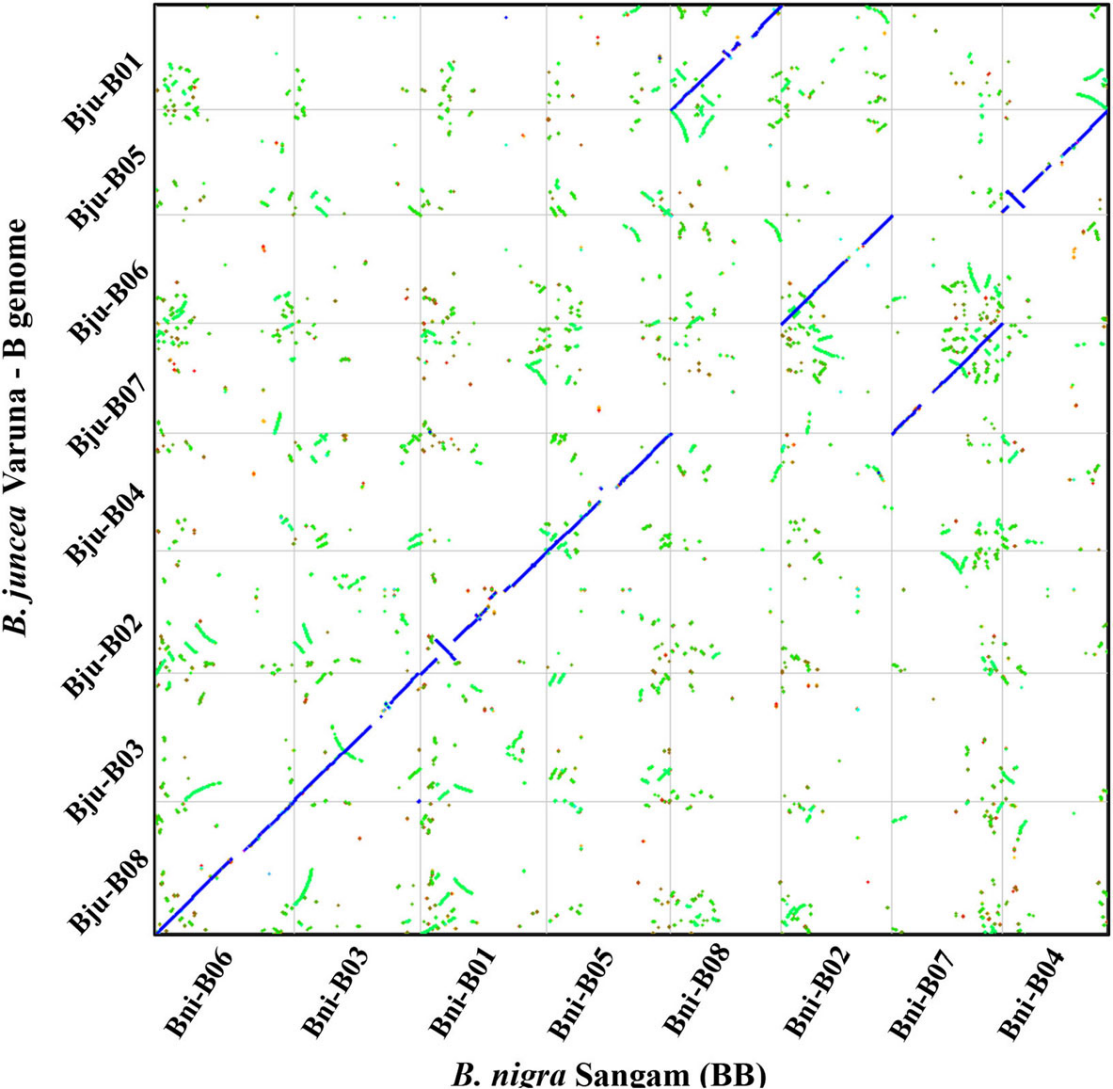
Comparison of *B. nigra* (BniB) pseudochromosomes with *B. juncea* B genome (BjuB) pseudochromosomes. The comparison was carried out with the Synfind program available at the CoGe website. Gene pairs with the least Ks value were identified as orthologous genes between the two genomes. Strictly orthologous genes have been denoted as blue dots, other syntenic regions are shown with the green dots. Very high gene collinearity was observed between the two B genomes, except for the three inversions in the *B. nigra* pseudochromosomes - BniB01, BniB04, and BniB08. Centromeric regions are devoid of genes and therefore, recognized as gaps. The nomenclature of the Bni pseudochromosomes is according to the new nomenclature, the BjuB pseudochromosome nomenclature is following Panjabi et al. [11]

We compared the overall genome architecture of the BniB and BjuB genomes by MCScanX based analysis. Orthologous genes were identified as the syntenic gene pairs having the least Ks value amongst all the possible combinations. The homologous gene pairs between the two B genomes were plotted using the Synmap analysis [25]. Very high collinearity was observed between the BniB and the BjuB pseudochromosomes (Fig. 2). An inversion was observed in each of the three pseudochromosomes – BniB01, BniB04, and BniB08 vis-à-vis the corresponding BjuB pseudochromosomes. The inversions in the BniB01 and BniB08 pseudochromsomes were found to be intra-block inversions in the U and F gene blocks, respectively. An inter-paleogenome non-contiguous gene block association [23] J_MF1_-I_MF1_-S_MF2_-S_LF_ observed in BjuB04 and shared with BraA04 and BolC04 was found to be J_MF1_-I_MF1_- J_MF1_-I_MF1_-S_MF2_-S_LF_ in BniB04. This new gene block association in BniB04 is due to an inversion in the J_MF1_-I_MF1._ This inversion seems to be specific to the sequenced Sangam genome. It can be concluded that the progenitor B genome of *B. juncea* did not contain all three inversions.

### New nomenclature for *B. nigra* pseudochromosomes

Highly contiguous pseudochromosome level assemblies have been available for *B. rapa* (BraA) [21], and *B. oleracea* (BolC) [22]; such an assembly is now available for *B. nigra* (BniB) allowing a chromosome level homology analysis.

**Fig. 3:**
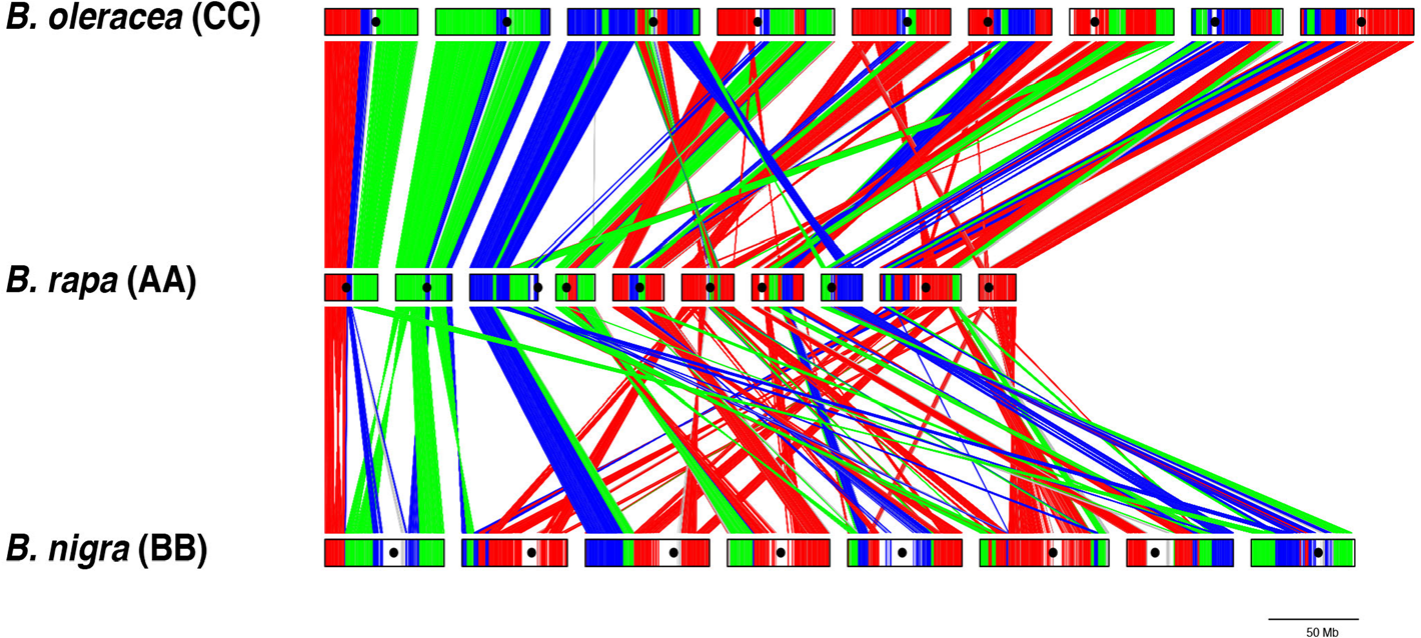
Comparative gene block arrangements in *B. rapa* [21], *B. nigra* (this study), and *B. oleracea* [22]. All the three assemblies are with long-read sequences. The LF, MF1 and MF2 paleogenomes present in the A, B and C genomes have been represented by red, green and blue colors, respectively. The A and C genomes show more similarity in gene block arrangements, whereas the B genome has divergent arrangements. The B genome pseudochromosomes are as per the new nomenclature based on maximum gene to gene collinearity with the *B. rapa* pseudochromosomes

We carried out such an analysis for the BraA and BniB pseudochromosomes keeping the nomenclature given to the BraA [13] pseudochromosomes as settled as it was the first sequenced genome from the U’s triangle. Each assembled pseudochromosome of *B. nigra* showed homology with more than one pseudochromosome of *B. rapa* (Fig. 3, Supplementary Fig. 8). The size of the genomic stretches from the BraA pseudochromosomes showing homology with different BniB pseudochromosomes was calculated (Table 2). Each BniB pseudochromosome was given the number of the BraA pseudochromosome with which it shared maximum homology (except pseudochromosome BniB02). As *B. nigra* has eight chromosomes against ten in *B. rapa*, homology with BraA09 and BraA10 was not taken into consideration. The new nomenclature is Version 3.

**Table 2:** The size of the genomic stretches from the *B. rapa* pseudochromosomes showing gene collinearity-based homology with different *B. nigra* pseudochromosomes. Colored boxes represent the new nomenclature V3 for *B. nigra*

		***B. nigra***							
	**BniB - V1**^a^	**B01**	**B06**	**B02**	**B07**	**B04**	**B05**	**B08**	**B03**
	**BjuB - V2**^a^	**B02**	**B06**	**B03**	**B05**	**B04**	**B08**	**B07**	**B01**
	**BniB - V3**^a^	**B01**	**B02**	**B03**	**B04**	**B05**	**B06**	**B07**	**B08**
***B. rapa*** **V3.0**	**A01**	15185553^b^ **51.3%**							13228780 **44.7%**
	**A02**	16418817 **52.2%**	14535932 **46.2%**						
	**A03**			15196210 **39.9%**			14502023 **38%**	1218940 **3.2%**	4307164 **11.3%**
	**A04**				14898239 **67.9%**	5900409 **26.9%**		702255 **3.2%**	
	**A05**				10591962 **37.2%**	13872273 **48.7%**		3293639 **11.5%**	
	**A06**		7524595 **19.1%**	5158622 **17.6%**		7315564 **24.3%**	8704851 **29.8%**		
	**A07**				2818377 **9.7%**	7270483 **25.23%**		15688850 **54.5%**	
	**A08**							11410503 **49.7%**	11463772 **49.9%**
	**A09**		21384087 **47.3%**	10792098 **23.9%**		126317 **0.27%**	2342318 **5.18%**	1203054 **2.7%**	3813925 **8.4%**
	**A10**		2004504 **9.7%**			2508933 **12.1%**	14954862 **72.1%**	887503 **4.2%**	

^a^V1- BniB LG nomenclature by Lagercrantz *et al*. [26] based on genetic mapping; V2- BjuB LG nomenclature by Panjabi *et al*. [11] based on genetic mapping; V3- BniB pseudochromosome nomenclature proposed in this study based on the long-read genome assembly

^b^Explanation of the numbers - As an example – pseudochromosome A01 of *B. rapa* has homology with two B genome chromosomes; a region of 15,185,553 bp (51.3% of the total length of A01) with one of the B genome pseudochromosome and a region of 13,228,780 bp (44.7% of the total length of A01) with the other

The current nomenclature (Version 1) for the *B. nigra* LGs, recommended by the internationally agreed standard (http://www.brassica.info), is based on some early work on the comparative genetic mapping between At and *B. nigra* [26]*.* A total of 160 DNA fragments from the At genome, mostly anonymous and some cDNA fragments of known genes, were used as RFLP markers. We carried out a more extensive mapping work on the A and B genomes of *B. juncea* using intron length polymorphism (IP) markers derived from the At genome [11]. This allowed a more extensive comparative genetic mapping between the A and the B genomes of *B. juncea* vis-a-vis the gene block organization in the At genome. A different nomenclature (Version 2) was suggested for the BjuB genome LGs based on the extent of homology with the BjuA LGs. This nomenclature was supported by genetic mapping in *B. juncea* using RNAseq based SNP markers [27].

While Version 1 and Version 2 are based on genetic mapping, Version 3 proposed in this study is based on gene collinearity and is, therefore, more accurate (Table 2). Version 1, due to low marker density is the most inaccurate. In Version 1- BniB02 and BniB05 have no homologous regions with BraA02 and BraA05 chromosomes, respectively. Version 2 is more accurate; however, in this version, BniB08 has no homology with BraA08. The inter-paleogenome non-contiguous gene block association J_MF1_-I_MF1_-S_MF2_-S_LF,_ which is evidence for a common origin of the A, B, and C genomes [23], is only accounted for in Version 3.

## Discussion

*B. nigra* genome assembly reported here is an improvement over the previous *B. nigra* assemblies that were based on short-read sequencing [16, 28]. The long-read ONT sequencing and optical mapping have provided a highly contiguous genome assembly, with five of the eight pseudochromosomes represented by a single scaffold. The centromeric and telomeric regions could also be identified. Recently, genome assemblies of two more lines of *B. nigra* – Ni100 and CN115125 have been reported using the ONT technology [29]. The N50 value of the assembled scaffolds of all the three ONT assemblies are quite similar, showing high contiguity of the assemblies. However, the genome size of the three sequenced *B. nigra* lines seem to be significantly different. We have estimated the *B. nigra* Sangam genome size to be ~ 522 Mb; the genome size of *B. nigra* Ni100 has been estimated to be ~ 570 Mb and that of line CN115125 to be ~ 608 Mb. An earlier study on *B. nigra* [28] estimated the genome size to be ~ 534 Mb. It will be interesting to compare the three ONT based chromosome level assemblies for the overall gene content and *B. nigra* specific genes.

*B. nigra* germplasm could be an important source for some of the major diseases afflicting the more extensively cultivated Brassica species. So far extensive efforts have been devoted to the transfer of resistance to the blackleg disease (causal organism *Leptosphaeria maculans*) from *B. nigra* to *B. napus* [30]. While the chromosomes of *B. nigra* containing resistance were identified in the chromosome addition lines [31], actual introgression has been difficult due to limited pairing between the B, and the A and C genome chromosomes [32]. This lack of pairing, in all probability, is due to a very divergent chromosomal organization between the B, and A/C genomes. Genetic exchanges may also be limited due to a strong mechanism in the B genome for suppression of pairing between the homeologous chromosomes [33, 34].

We have compared the *B. nigra* (BniB) genome assembly reported in this study with the B genome of *B. juncea* (BjuB) assembled with SMRT sequencing and optical mapping [23], and shown that the two genomes are collinear in gene arrangement, and have similar gene content and centromeric structures. We have earlier shown that the A genome of *B. juncea* (BjuA) [23] is similar to the *B. rapa* (BraA) [21] genome. The success of the natural allotetraploid *B. juncea* was therefore based on immediate stability due to suppression of homoeologous pairing between the A and the B genome as has been suggested in some of the early cytogenetic studies [28, 30]. However, high collinearity between BniB and BjuB genomes would allow the use of *B. nigra* germplasm for broadening the genetic base of *B. juncea* and transfer of disease resistance and other traits from *B. nigra* to *B. juncea*. As an example, *B. nigra* line 2782 is resistant to a number of isolates of oomycete pathogen *Albugo candida* and can be a useful source of resistance for the susceptible Indian gene pool lines of *B. juncea* [35].

We have suggested a new nomenclature for the *B. nigra* LGs/chromosomes. The nomenclature currently in use does not follow any structural or evolutionary relationship with the other Brassica species of the U’s triangle. Any nomenclature should reflect some evolutionary relationships. The new nomenclature reflects the extent of homology between the B genome and the A and C genomes. As more species belonging to the tribe Brassiceae are sequenced, it would be useful to take the *B. rapa* LG/pseudochromosome nomenclature as the baseline for assigning nomenclature to the pseudochromosomes of the newly sequenced species, as we have done in the case of *B. nigra*. We propose that the suggested nomenclature for the B genome LGs/chromosomes be accepted by the Brassica researcher community.

## Conclusion

We report a highly contiguous genome assembly of *B. nigra* (BB) variety Sangam using Oxford Nanopore long reads and optical mapping. Five of the eight chromosomes are represented by one scaffold each. The assembled genome of ~ 505.18 Mb contains ~ 246 Mb of repeat elements and 57,249 protein-encoding genes. Transcriptome analysis validated 42,444 of the predicted genes. A comparison of the A, B, and C genomes of the three diploid species of the U’s triangle showed the B genome to be divergent from the A and C genomes in the gene block arrangements and the centromeric regions. A comparison of the B genomes of *B. juncea* (AABB) and *B. nigra* (BB) showed a highly collinear gene arrangement between the two genomes. We propose a new nomenclature for the B genome pseudochromsomes based on maximum homology with the A genome pseudochromosomes.

## Methods

### Plant material, genome size estimation, nanopore sequencing, optical mapping, and genome assembly

A DH (doubled haploid) line BnSDH-1 of *Brassica nigra* variety Sangam [23; NCBI BioSample id: SAMN05210941] was used for genome sequencing and assembly. BnSDH-1 was maintained by bud pollination. For DNA isolation, BnSDH-1 seedlings were grown in a growth chamber maintained at 8 h light, 25 °C / 16 h dark, 10 °C cycle. DNA was isolated from the leaves of 10 d old seedlings; the harvested leaves were immediately frozen in liquid nitrogen. High molecular weight DNA was isolated from the leaf tissues by the CTAB method [36]. For Nanopore sequencing, genomic DNA libraries were prepared using the ‘Ligation sequencing kit 1D’ following the manufacturer’s instructions (Oxford Nanopore). In brief, around 2 μg of high molecular weight DNA was repaired using the ‘NEBNext FFPE DNA Repair mix’ and the ‘Ultra ll End-prep Enzyme mix’; subsequently, the adapter mix was ligated to the repaired DNA using the ‘NEBNext Quick T4 DNA Ligase’. At the end of each step, DNA was cleaned with the ‘AMPure XP beads’ (Thermo Fisher Scientific). The quality and quantity of the DNA libraries were determined with a Nanodrop spectrophotometer. DNA libraries were sequenced on the MinION device using the MinION Flow Cells R 9.4.1 (Oxford Nanopore). Base-calling and quality filtering were carried out using Albacore software (v2.5.11; https://github.com/Albacore). Illumina short-read sequencing data (~100x coverage) [23] of the line BnSDH-1 was used at various steps (described wherever used) of the new genome assembly. Approximately 40x Illumina PE (2 × 100 bp) data with a kmer length of 21 was used for the kmer frequency distribution analysis with Jellyfish v2.2.6 [37]. The output histogram file was used to estimate the genome size of BnSDH-1 using the findGSE program [38].

Raw Nanopore reads were assembled into contigs using the Canu assembler v1.6 [39] with the parameters ‘minRead length’ and ‘minOverlap length’ set at values of 1000 bp. The paired-end (PE) reads obtained earlier with Illumina sequencing (~100x coverage) were mapped on the assembled Nanopore contigs using BWA-MEM (v0.7.12) [40], followed by error correction with the Pilon (v1.23) program [41] in five iterative cycles. After each of the Pilon cycles, completeness of the corrected genome was ascertained with Benchmarking Universal Single Copy Orthologue (BUSCO) program (v4.0.5) [42]. OrthoDB v10 plant datasets were used as the reference for analyzing the completeness of the predicted genes.

Optical mapping was carried out following the protocols suggested by the manufacturer (Bionano Genomics). Leaf tissues from 7 d old seedlings were harvested and transferred to an ice-cold fixing solution. Nuclei were isolated using the ‘rotor-stator’ protocol (Bionano Genomics, Document no: 30228) and the nuclear fraction was purified on a sucrose density gradient. The nuclei were embedded in 0.5% w/v agarose followed by treatment with proteinase-K (Qiagen) for 2 h. Mapping was carried out with two different labeling reactions – one NLRS (Nick Label Repair and Stain), and one DLS (Direct Label and Stain). For the NLRS labeling reaction, agarose plugs were treated with the enzyme *Bss*SI, and the nicks were labeled with the ‘IrysPrep NLRS labeling kit’. In the DLS labeling reaction, DNA was recovered from the agarose plugs, suspended in TE buffer, and labeled with the ‘Bionano Prep DLS kit’. Mapping data were obtained from the labeled libraries on the Saphyr system (Bionano) using one lane for each library. Mapping and hybrid assemblies were performed using the Bionano Access software (V1.5.2).

A previously generated genetic map of *B. nigra* [23], developed using an F_1_DH population from a cross of line BnSDH-1 × line 2782 was used for validating the scaffold level assemblies and assigning the scaffolds to the eight linkage groups (LGs) to constitute eight pseudochromosomes. The position of the GBS marker tags was determined on the scaffolds with a Blastn search analysis. A correlation plot of the physical and genetic position of the markers was developed to validate the integrity and quality of scaffolding. Scaffolds were positioned and oriented on each pseudochromosome based on the information obtained with the correlation plot.

### Transcriptome sequencing, gene, and transposon annotation

Illumina short-read based transcriptome sequencing of the line BnSDH-1 has been reported earlier [23]. The transcriptome sequencing of the line 2782, an East European gene pool line of *B. nigra* was performed in this study (Supplementary File 2). Along with these, a PacBio based IsoSeq sequencing of the line BnSDH-1 was also carried out. For the PacBio based transcriptome sequencing, total RNA was isolated from the seedling, leaf, and developing inflorescence tissues using the ‘Spectrum plant total RNA kit’ (Sigma). The quality of the RNA was checked with Bioanalyzer 2100 using the ‘RNA 6000 Nano kit’ (Agilent). RNA samples with RIN values >7 were used for further analysis. Transcriptome sequencing was carried out on the pooled RNA. Three different libraries of the size range – 0.5 – 1 kb, 1 – 2 kb, and 2 – 6 kb were prepared using the ‘SMRTbell Template Prep kit’ and sequenced on a PacBio RS II sequencer. The raw sequences obtained from each of the three libraries were assembled separately using SMRT Analysis software (v1.4). Full-length non-chimeric sequences were used for clustering with ICE (Structure Clustering and Error Correction) algorithm; partial reads were used for polishing of the ICE generated consensus sequences. ORFs were predicted from the polished consensus sequences using the ANGEL software (https://github.com/PacificBiosciences/ANGEL).

Transposable elements (TEs) were identified in the genome assembly using the Repeatmodeler pipeline (http://www.repeatmasker.org/RepeatModeler/). A *de-novo* repeat library was developed using RECON (v1.0.5), RepeatScout, and Tandem Repeat Finder programs available in the Repeatmodeler pipeline, and NSEG (ftp://ftp.ncbi.nih.gov/pub/seg/nseg/) program. The developed TE library, along with the repbase database for At was used to predict TEs in the assembled genome using RepeatMasker (htntp://www.repeatmasker.org). Identified LTR sequences were validated by the LTR finder program (v 1.0.2) [43].

For gene annotation, repeat-masked genome assembly was used to predict the protein-coding genes with the Augustus program (v3.2.1) [24] trained with 250 randomly selected *B. rapa* genes as the reference data set. The predicted genes were validated by a blast search against the Uniprot protein database (e value threshold <1e-05). The predicted genes were validated by mapping the previously generated Illumina RNA-seq sequences [23], and the RNA-seq and Iso-seq sequences generated in this study on the assembled genome. Illumina RNA-seq reads were mapped with the STAR aligner (v 2.5.3a) [44] and Iso-seq sequences were mapped using the Minimap2 program [45] using the default parameters.

### Syntenic block identification and determination of gene fractionation patterns

Syntenic regions in the assembled genome were identified with the MCScanX program [46]. An all-against-all Blastp comparison was carried out between the *B. nigra* assembly and previously reported BraA [21], BjuA, and BjuB [23], and At genome assemblies (e-value threshold 1e-05). The blastp output file was used along with the information of positions of each gene in all the genomes for synteny analysis. Parameters for the MCScanX were set as match_score: 50, match_size: 5, gap_penalty: − 1, e-value: 1e-05, max_gaps: 25. Genes retained in each of the syntenic regions were calculated in a sliding window of 500 flanking genes at a given locus of At.

For divergence analysis, DNA sequences and the protein sequences of At genes and their orthologs in the BraA, BniB, and BjuB genomes were aligned with MUSCLE v3.8.31 software [47]. Poorly aligned regions were trimmed using GBLOCKS (v0.91) [48] and PAL2NAL scripts. A custom Perl script was used for the conversion of the aligned fasta format to the Phylip format. The Phylip files were converted into a Newick format tree, and the Ks values were obtained using the PAML package.

## Supplementary Information

**Additional file 1 : Supplementary File 1.** *Brassica. nigra* genome assembly – Bionano optical mapping stats.

**Additional file 2: Supplementary File 2.** RNA sequencing studies in *Brassica nigra*.

**Additional file 3: Supplementary Table 1.** *Brassica nigra* variety Sangam raw sequencing data obtained with Oxford Nanopore machine. **Supplementary Table 2a.** Position and number of the assembled scaffolds and contigs on each of the eight pseudochromosomes of *Brassica nigra*. **Supplementary Table 2b.** Statistics of some of the recent genome assemblies of A, B, and C genomes of Brassiceae tribe. **Supplementary Table 3.** *Brassica nigra* Sangam genome – types of TEs and other repeats.

**Additional file 4: Supplementary Figure 1.** K-mer frequency distribution in ~40x PE Illumina reads of *B. nigra* line BnSDH-1. The frequency of the kmers of length 21bp was calculated and used to estimate the genome size with FindGSE program. The *B. nigra* genome was estimated to be ~522.13 Mb in size. **Supplementary Figure 2.** Correction of *B. nigra* Nanopore assembly contigs with ~100x Illumina short-reads using the Pilon program. Five rounds of Pilon based corrections of SNPs and InDels were carried out iteratively. Most of the errors were identified and corrected in the first two cycles. Green bars represents the BUSCO score of complete gene models achieved after each round of correction. **Supplementary Figure 3.** Relationship between the GBS markers on the genetic map of *Brassica nigra* Sangam x 2782 F1DH population [23] and physical position of the respective marker tags on the *B. nigra *Sangam genomic sequences. Genetic positions of the markers have been shown on the xaxis and position of the marker in the assembled genome on the y-axis. A linear relationship was found between the physical and genetic distances of the markers present on the LGs. The centromeric regions showed lower rate of recombination as compared to other regions of the chromosomes. **Supplementary Figure 4.** Distribution of different TEs on the *B. nigra* pseudochromosomes. LTR/Copia and LTR/Gypsy type transposable elements are the most abundant TEs. Centromeric regions show a much higher content of LTR/Copia TEs. **Supplementary Figure 5.** Distribution of the B genome-specific centromeric repeats on the eight pseudochromosomes of *B. nigra*. The earlier described six unique repeat sequences in the B genome of *B. juncea* [23] were found to constitute the centromeric regions of the B. nigra pseudochromosomes. The position of the centromeric repeats on the pseudochromosomes has been shown by horizontal bars; the vertical curve represents the cumulative number of the predicted centromeric repeats. **Supplementary Figure 6.** Genes identified in the transcriptome of leaf, stem, and developing inflorescence of B. nigra. Genes present on the eight pseudochromosomes of B. nigra are represented in track 1; each gene is represented by a line and colored based on the gene bock it belongs to, following Schranz et al. [17].. Track 2 represents the position of the centromeric repeats in each of the *B. nigra* pseudochromosomes. Track 3 represents the position of the genes found to be expressed in the transcriptome sequencing data; each expressing gene has been represented by a green line; genes nonexpressing in the transcriptome study are represented by red lines. **Supplementary Figure 7.** Orthologous gene retention in the BraA [21], BniB (this study), and BolC [22] genomes corresponding to the *A. thaliana* genes. Position of the *A. thaliana* genes have been plotted on axis X, the proportion of the genes retained in each of the three constituent paleogenomes of the A, B and C genomes has been plotted on axis Y. The constituent paleogenomes have been designated LF (least fragmented), MF1 (moderately fragmented), and MF2 (most fragmented) based on the percentage of genes retained in comparison to At, following the convention set for *B. rapa* [13]. **Supplementary Figure 8.** Comparison of the eight *B. nigra* (BniB) pseudochromosomes with the ten *B. rapa* (BraA) pseudochromosomes [21] for homologous regions. Each of the horizontal lines represents a gene. Homologous regions were identified by gene collinearity and the least Ks values amongst all the possible gene pairs. The number given to each B genome pseudochromosome in most of the cases is based on the number given to the A genome pseudochromosome with which it shows maximum homology.

**Additional file 5: Supplementary Table 4.** Genes predicted on different pseudochromosomes of *Brassica nigra* Sangam and their orthologs in *Arabidopsis thaliana* (along with their respective gene blocks) and *B. juncea* Varuna B genome (BjuB) and *B. rapa* Chiifu V3.0 (BraA) genome. Column A – gene blocks as identified in *A. thaliana*; Column B – *A. thaliana* orthologs with gene id; Column C – paleogenome of *B. nigra* to which the gene belongs; Column D – predicted *B. nigra* gene id; Column E – physical position of the genes on the pseudochromosomes; Column F – expression status of the predicted *B. nigra* genes (“Expressed” means that the gene was found in the transcriptome analysis in this study or other studies described in **Supplementary File 2**, “Not expressed” represents – expression not found); Column G – *B. juncea* B genome (BjuB) orthologs with the gene id; Column H –*B. rapa* V3.0 (BraA) orthologs with the gene id.

## Abbreviations

Bni: *Brassica nigra*
CTAB: Cetyl trimethylammonium bromide
