# Supplementary material for: A highly contiguous genome assembly of Brassica nigra (BB) and revised nomenclature for the pseudochromosomes
Source: BMC Genomics. 2020 Dec 11;21:887. doi: 10.1186/s12864-020-07271-w (PMC7731534; doi:10.1186/s12864-020-07271-w)
Supplement: Supplementary file 1 — Additional file 1 : Supplementary File 1. Brassica. nigra genome assembly – Bionano optical mapping stats. [file 12864_2020_7271_MOESM1_ESM.docx]

**Supplementary File 1**

***B. nigra* genome assembly – Bionano optical mapping stats**

This file contains the machine generated report on the data generated with two optical maps – one with NLRS technology using a *Bss*SI library and the other with DLS method using DLE I enzyme. The file also contains a table summarizing the input data and outputs at each step of optical mapping for scaffolding the contigs generated by ONT and corrected by Pilon using Illumina short-reads.

1. **Machine generated report for the hybrid assembly**

**Automatic Conflicts Detections from BNG-NGS alignment:**

Number of conflict cuts made to Bionano maps (CTTAAG): 3

Number of conflict cuts made to Bionano maps (CACGAG): 3

Number of conflict cuts made to NGS sequences: 204

Number of BNG maps to be cut (CTTAAG): 3

Number of BNG maps to be cut (CACGAG): 3

Number of NGS contigs to be cut: 148

**Input NGS sequence statistics:**

Count = 1549

N50 length (Mbp) = 1.509

Total length (Mbp) = 520.090

**Input Bionano Genome map statistics (CTTAAG) DLE I**

Count = 17

N50 length (Mbp) = 63.486

Total length (Mbp) = 567.374

**Input Bionano Genome map statistics (CACGAG) *Bss*SI**

Count = 440

N50 length (Mbp) = 1.683

Total length (Mbp) = 522.224

**Hybrid scaffold stats:**

Total number of hybrids: 17

Two-enzyme hybrid scaffold N50 (Mbp): 70.240

Scaffold N50 of final hybrids inclusive of single-enzyme hybrids (Mbp): 70.240

Total length of hybrid scaffolds (Mbp): 509.637

Total length of unscaffoled NGS (Mbp): 30.435

Longest hybrid (Mbp): 115.469

Total Numbers of NGS anchored in final hybrids: 753

Anchored in two-enzyme hybrid: 749

Anchored in single-enzyme hybrid1 only: 2

Anchored in single-enzyme hybrid2 only: 3

Total NGS not in scaffold: 1051

Total length of NGS anchored in final hybrids (Mbp): 489.654

N50 of sequence anchored (Mbp): 1.506

Anchored with two-enzyme (Mbp): 489.005128

Anchored with single-enzyme hybrid1 only (Mbp): 0.397

Anchored with single-enzyme hybrid2 only (Mbp): 0.445

Total NGS not in scaffold (Mbp): 30.435

N50 of sequence not in scaffold (Mbp): 0.036

Scaffold N50 of final hybrids inclusive of unscaffolded NGS (Mbp): 64.585

**Stats calculated from final FASTA file:**

Total number of hybrid (Mbp): 15

Hybrid scaffold n50 (Mbp): 70.405

Scaffold N50 of final hybrid inclusive of unscaffolded NGS sequences (Mbp): 64.11

Total length of hybrid scaffold (Mbp): 506.396

Total length of unscaffoled NGS (Mbp): 30.435

1. **Summary of the generated data**

| **Enzyme** | **No. of maps** | **Average map length (Mb)** | **Coverage (mol > 150 Kb)^*^** | **Assembly size (Gb)** | **N50 value (Mb)** | **Label Density (/100 Kb)** |
| --- | --- | --- | --- | --- | --- | --- |
| **Input NGS Assembly** | 1549 |  |  | 520 | 1.5 |  |
| **BssSI De novo** | 440 | 522.24 | 177.58 | 522.24 | 1.68 | 14.51 |
| **DLE-1** | 17 | 567.3 | 134.5 | 567.3 | 63.48 | 14.32 |
| **DLE-1 and BssS1 NGS Hybrid** | 15 |  |  | 506.39 | 70. 4 |  |
