# Supplementary material for: A highly contiguous genome assembly of Brassica nigra (BB) and revised nomenclature for the pseudochromosomes
Source: BMC Genomics. 2020 Dec 11;21:887. doi: 10.1186/s12864-020-07271-w (PMC7731534; doi:10.1186/s12864-020-07271-w)
Supplement: Supplementary file 3 — Additional file 3: Supplementary Table 1. Brassica nigra variety Sangam raw sequencing data obtained with Oxford Nanopore machine. Supplementary Table 2a. Position and number of the assembled scaffolds and contigs on each of the eight pseudochromosomes of Brassica nigra. Supplementary Table 2b. Statistics of some of the recent genome assemblies of A, B, and C genomes of Brassiceae tribe. Supplementary Table 3. Brassica nigra Sangam genome – types of TEs and other repeats. [file 12864_2020_7271_MOESM3_ESM.docx]

**Supplementary Table 1**. ***Brassica nigra* variety Sangam raw sequencing data obtained with Oxford Nanopore machine**

| **PacBio raw reads** | **stats** |
| --- | --- |
| Number of reads | 8,778,822 |
| Total size of reads (bp) | 52,227,562,135 |
| Longest read | 182,926 |
| Number of reads > 1K nt | 7,608,368 |
| Number of reads > 10K nt | 1,558,964 |
| Number of reads > 100K nt | 136 |
| Mean read size | 5,949 |
| Median read size | 3,816 |
| N50 read length | 10,090 |
| L50 read count | 1,536,070 |

**Supplementary Table 2a**. **Position and number of the assembled scaffolds and contigs on each of the eight pseudochromosomes of *Brassica nigra***

| **S. No** | **Chromosome** | **Number of scaffolds/**  **contigs** | **Scaffolds name** | **Length (bp)** | **Features** |
| --- | --- | --- | --- | --- | --- |
| 1 | BniB01 | 1 | Scaffold_4a | 56,519,733 | Centromere |
| 2 | BniB02 | 3 | Scaffold_7 | 48,531,046 | Telomere, Centromere |
|  |  |  | Scaffold_100000100016 | 203,637 |  |
|  |  |  | Scaffold_8 | 18,041,172 | Telomere |
| 3 | BniB03 | 2 | Scaffold_5 | 68,578,869 | Telomere, Centromere |
|  |  |  | Scaffold_12 | 899,017 |  |
| 4 | BniB04 | 4 | Scaffold_13 | 784,688 | Telomere |
|  |  |  | Scaffold_9 | 15,344,140 |  |
|  |  |  | Scaffold_10 | 4,249,561 |  |
|  |  |  | Scaffold_3 | 43,609,976 | Telomere, Centromere |
| 5 | BniB05 | 1 | Scaffold_6 | 57,438,084 | Telomere, Centromere |
| 6 | BniB06 | 1 | Scaffold_4b | 59,096,763 | Telomere, Centromere |
| 7 | BniB07 | 1 | Scaffold_1 | 59,653,443 | Centromere |
| 8 | BniB08 | 1 | Scaffold_2 | 72,232,902 | Telomere, Centromere |
|  | Other scaffolds | 1 | Scaffold_200000101 | 498,801 | Chloroplast |

**Supplementary Table 2b**. **Statistics of some of the recent genome assemblies of A, B, and C genomes of Brassiceae tribe**

|  | ***B. rapa* V3.0** | ***B. oleracea*** | ***B. nigra*** | ***B. nigra*** | ***B. nigra*** |
| --- | --- | --- | --- | --- | --- |
| **Study** | Zhang et al., 2018 | Belsar et al., 2018 | Yang et al., 2016 | Wang et al., 2019 | This study |
| **Sequencing method** | PacBio | ONT | Illumina | Illumina | ONT |
| **Estimated genome size** | 455 Mb | 630 Mb | 591 Mb | 534 Mb | 522 Mb |
| **Genome Assembled** | 353.14 | 554.9 Mb | 396.9 Mb | 512 Mb | 515.4 Mb |
| **Number of Contigs** | 1498 | 264  (≥ 500bp) | 25,103 | 116,346 | 1,549 |
| **N50 contigs** | 1.45 Mb | 9.4 Mb | 0.31 Mb | 0.18 Mb | 1.4 Mb |
| **Scaffolds** | 1,301 | 140  (>2 Kb) | 5,120  (>1 Kb) | 75,048 | 15 |
| **N50 scaffolds** | 4.4 Mb | 29 Mb | 0.56 Mb | 0.16 Mb | 68.5 Mb |
| **Total genome assembled in pseudochromosomes** | 298.19 Mb | 528.8 | 136.8 Mb | 409.76 Mb | 505.2 Mb |
| **Percentage of genome in pseudochromosome** | 65.5 % | 87.9% | 66% | 76.5 % | 96.7 % |

**Supplementary Table 3. *Brassica nigra* Sangam genome – types of TEs and other repeats**

|  | **TE type** | **Repeat elements** | **Intact copies** | **Length** | **% genome coverage** |
| --- | --- | --- | --- | --- | --- |
| **Class I: DNA transposon** | DNA/CMC-EnSpm | 12933 | 11866 | 8896721 | **1.77** |
|  | DNA/Crypton-S | 1916 | 1701 | 690761 | **0.14** |
|  | DNA/hAT | 2601 | 2358 | 1270894 | **0.25** |
|  | DNA/hAT-Ac | 16363 | 15067 | 5949507 | **1.18** |
|  | DNA/hAT-Charlie | 863 | 849 | 482279 | **0.1** |
|  | DNA/hAT-Tag1 | 5442 | 5019 | 1509057 | **0.30** |
|  | DNA/hAT-Tip100 | 994 | 861 | 407834 | **0.08** |
|  | DNA/IS3EU | 296 | 286 | 107212 | **0.02** |
|  | DNA/Maverick | 157 | 155 | 52930 | **0.01** |
|  | DNA/Merlin | 44 | 33 | 11427 | **0.002** |
|  | DNA/MuLE-MuDR | 6156 | 5030 | 4456749 | **0.88** |
|  | DNA/PIF-Harbinger | 7263 | 6632 | 2467504 | **0.49** |
|  | DNA/RC | 4419 | 3526 | 1981015 | **0.39** |
|  | DNA/TcMar-Pogo | 3289 | 3063 | 756166 | **0.15** |
|  | DNA/TcMar-Stowaway | 7037 | 6912 | 1451632 | **0.29** |
|  | DNA/Zisupton | 1467 | 1304 | 412805 | **0.082** |
| **Subtotal** |  | **71240** | **64662** | **30904493** | **6.13** |
| **Class II: Retrotransposon** | LINE/L1 | 15495 | 14134 | 8975037 | **1.78** |
|  | LINE/Penelope | 923 | 879 | 295226 | **0.059** |
|  | LTR/Cassandra | 1560 | 1516 | 584873 | **0.12** |
|  | LTR/Caulimovirus | 987 | 947 | 605419 | **0.12** |
|  | LTR/Copia | 35484 | 29662 | 43630011 | **8.66** |
|  | LTR/DIRS | 233 | 230 | 76735 | **0.01** |
|  | LTR/ERV1 | 52 | 33 | 22473 | **0.005** |
|  | LTR/Gypsy | 65586 | 57202 | 103103094 | **20.46** |
|  | LTR/Pao | 162 | 134 | 31273 | **0.006** |
|  | SINE? | 124 | 124 | 7320 | **0.001** |
|  | SINE/tRNA | 3718 | 3687 | 562360 | **0.11** |
| **Subtotal** |  | **124324** | **108548** | **157893821** | **31.33** |
| **Other Repeats** | **Satellites** | **557** | **NA** | **164157** | **0.26** |
|  | **Simple repeats** | **1591** | **NA** | **577409** | **0.92** |
|  | **Unknown** | **160773** | **NA** | **59057277** | **9.38** |
|  |  |  |  |  |  |

**Supplementary Table 4. Genes predicted on different pseudochromosomes of *Brassica nigra* Sangam and their orthologs in *Arabidopsis thaliana* (along with their respective gene blocks) and *B. juncea* Varuna B genome (BjuB) and *B. rapa* Chiifu V3.0 (BraA) genome.** Column A – gene blocks as identified in *A. thaliana*; Column B – *A. thaliana* orthologs with gene id; Column C – paleogenome of *B. nigra* to which the gene belongs; Column D – predicted *B. nigra* gene id; Column E – physical position of the genes on the pseudochromosomes; Column F – expression status of the predicted *B. nigra* genes (“Expressed” means that the gene was found in the transcriptome analysis in this study or other studies described in Supplementary File 1, “Not expressed” represents – an expression not found); Column G – *B. juncea* B genomes (BjuB) orthologs gene id; Column H –*B. rapa* V3.0 (BraA) orthologs with the gene id
